# Role of hypoxia and glycolysis in the development of multi-drug resistance in human tumor cells and the establishment of an orthotopic multi-drug resistant tumor model in nude mice using hypoxic pre-conditioning

**DOI:** 10.1186/1475-2867-11-3

**Published:** 2011-02-14

**Authors:** Lara Milane, Zhenfeng Duan, Mansoor Amiji

**Affiliations:** 1 Department of Pharmaceutical Sciences, School of Pharmacy, Northeastern University, 360 Huntington Avenue, Boston, MA 02115, USA; 2 Department of Orthopaedic Surgery, Massachusetts General Hospital, Boston, MA 02114, USA; 3 Sarcoma Biology Laboratory, Center for Sarcoma and Connective Tissue Oncology, Massachusetts General Hospital, Boston, MA 02114, USA

## Abstract

**Background:**

The development of multi-drug resistant (MDR) cancer is a significant challenge in the clinical treatment of recurrent disease. Hypoxia is an environmental selection pressure that contributes to the development of MDR. Many cancer cells, including MDR cells, resort to glycolysis for energy acquisition. This study aimed to explore the relationship between hypoxia, glycolysis, and MDR in a panel of human breast and ovarian cancer cells. A second aim of this study was to develop an orthotopic animal model of MDR breast cancer.

**Methods:**

Nucleic and basal protein was extracted from a panel of human breast and ovarian cancer cells; MDR cells and cells pre-exposed to either normoxic or hypoxic conditions. Western blotting was used to assess the expression of MDR markers, hypoxia inducible factors, and glycolytic proteins. Tumor xenografts were established in the mammary fat pad of *nu/nu *mice using human breast cancer cells that were pre-exposed to either hypoxic or normoxic conditions. Immunohistochemistry was used to assess the MDR character of excised tumors.

**Results:**

Hypoxia induces MDR and glycolysis *in vitro*, but the cellular response is cell-line specific and duration dependent. Using hypoxic, triple-negative breast cancer cells to establish 100 mm^3 ^tumor xenografts in nude mice is a relevant model for MDR breast cancer.

**Conclusion:**

Hypoxic pre-conditiong and xenografting may be used to develop a multitude of orthotopic models for MDR cancer aiding in the study and treatment of the disease.

## 1. Introduction

### 1.1. Multi-Drug Resistance in Cancer

The development of multi-drug resistant (MDR) cancer is a challenge in the treatment of non-responsive, recurrent disease [1-6]. MDR refers to a state of resilience against structurally and/or functionally unrelated drugs; MDR can be intrinsic (innate) or acquired through exposure to chemotherapeutic agents [1].

The mechanisms of MDR include decreasing drug influx into a cell, increasing drug efflux out of a cell, increased DNA repair, increased drug metabolism/detoxification, and decreased apoptosis [7]. The most characterized mechanism of MDR is increased drug efflux through transmembrane pumps [7-9]. Over 13 ATP-Binding Cassette (ABC) transporters have been verified to contribute to MDR; of these, P-glycoprotein (Pgp) is the most consistently over-expressed and the most studied ABC transporter involved in the development of MDR cancer [8-10]. Membrane-bound Pgp effluxes a broad spectrum of substrates and active efflux requires the hydrolysis of two ATP molecules [7]. A recent study evaluating the cellular onset of MDR identified Pgp over-expression as the primary mechanism of MDR before malignant transformation [6]. Pgp over-expression is associated with poor prognosis in many types of cancer [7]. Other ABC transporters that contribute to MDR include multi-drug resistance protein 1 (MRP-1, ABCC1) and breast cancer resistance protein (BCRP, ABCG2) [9-12]. Additional proteins, such as growth factor receptors, are also used as markers of MDR; for example, over-expression of epidermal growth factor receptor (EGFR) is often associated with aggressive phenotypes and is used as a MDR marker in certain types of cancer [13-15].

### 1.2. Hypoxia and the Tumor Microenvironment

Perhaps the most significant contributor that defines the microenvironment of a tumor is the tumor vasculature [16-18]. The vascular network provides a tumor with oxygen and nutrients and is an avenue for the tumor to metastasize to remote sites. The importance of tumor vasculature is exploited by the plethora of anti-vasculature and anti-angiogenic cancer therapies [19,20]. Yet this vasculature is highly disorganized and constantly changing. Angiogenesis and vascular destruction are dynamic, ongoing processes; as the tumor is established new blood vessels are formed, this process continues as the tumor grows, but as the tumor propagates and expands blood vessels may be destroyed or cut off [16-18]. This haphazard process of neo- and de-vascularization contributes to the evolving phenotype of a tumor. A critical consequence of this fluctuation is a corresponding fluctuation in oxygen and glucose levels which results in heterogeneous states of hypoxia, anaerobic glycolysis (the Pasteur effect), and aerobic glycolysis (the Warburg effect) [17].

States of chronic hypoxia and transient hypoxia may occur and alter within the same tumor mass [21]. Chronic hypoxia occurs when a cell is beyond the diffusion limit of oxygen from a blood vessel (70-100 μm) whereas transient hypoxia occurs due to local oxygen depletion [21].

The cascade of proteome alterations that occurs in response to hypoxia begins with the transcription factor, Hypoxia Inducible Factor (HIF). HIF consists of alpha and beta subunits [22,23]. HIF-1α and HIF-1β are the most common isoforms; expression of HIF-2α and HIF-3α is more limited to healthy (non-cancerous) tissue [23].

Synthesis of the alpha subunit is oxygen independent while degradation is oxygen dependent [22,24]. Under conditions of hypoxia, the alpha subunit of HIF is stabilized and is then able to translocate to the nucleus [22,24,25]. Once localized to the nucleus, HIF-α forms a complex with HIF-β; this activated HIF complex is then able to bind to hypoxia responsive elements (HRE) on target genes inducing transcription [22,24].

Hypoxia has been shown to contribute to MDR and resistance to radiation therapy [17,21,24,26-29]. Some of the HIF-1α targets involved in MDR and aggressive cancer involve MDR1, matrix metalloproteinases (MMP), vascular endothelial growth factor (VEGF), VEGF receptor, transforming growth factor (TGF)-α, cyclooxygenase (COX)-2, nitric oxide synthase (NOS), insulin-like growth factor (IGF)-2, cathepsin D (CATHD), collagen type V (α1) [21,22,24,26,28-32]. HIF-1α also targets glycolytic proteins such as the glucose transporters GLUT-1 and GLUT-3, hexokinase 1 and 2, phosphofructokinase (PFK), aldolase, glyceraldehyde-3-phosphate dehydrogenase (GAPDH), phosphoglycerate kinase (PGK), enolase, pyruvate kinase, and lactate dehydrogenase (LDH) [21,22,24,26,28,29,32-36]. Reverse activation: glycolytic proteins activating HIF-1α, has also been demonstrated. This implies a possible feedback loop between the glycolytic and HIF-1α pathways that may be critical in the transformation of a normal cell into a cancer cell [34].

HIF is stabilized under hypoxia but also by many oxygen-independent factors such as epidermal growth factor receptor (EGFR), heat-shock protein 90, phosphatidylinositol 3-kinase, and cyclooxygenase-2 activity [24,37,38]. As such, HIF has been established to contribute to cancer cell glycolysis in both the absence and presence of oxygen (the Pasteur and Warburg Effects) [34,35,39-41].

### 1.3. The Pasteur and Warburg Effects

Traditionally, in the presence of oxygen cells obtain energy through oxidative phosphorylation (OXPHOS) and oxygen inhibits glycolysis; this inhibition is known as the Pasteur effect [39]. Some cancer cells also undergo aerobic glycolysis (the Warburg effect); this phenomenon was first discovered by Otto Warburg in 1930 [39,42-45]. As there is a constant flux in the oxygenation states of a solid tumor (between normoxic, hypoxic, and anoxic levels), increased glycolysis in both the absence and presence of oxygen are important hallmarks of cancer [34,35,39-41].

The biological motivation for increased glycolysis is multifold. First, glycolysis is much safer than oxidative phosphorylation. OXPHOS produces ROS which can be very damaging to the delicate balance of ROS maintained in an abnormal cancer cell; decreasing reliance on OXPHOS is a means of limiting ROS accumulation [39,46]. Secondly, although the net energy yield from glycolysis is much lower than OXPHOS (2 ATP verses 36), the process is much faster, providing a cell with direct energy acquired in the cytoplasm [39]. Third, a decreased reliance on OXPHOS ensures that oxygen does not become a limiting factor to survival [39,47]. Fourth, the glycolytic pathway provides precursors for biomolecules necessary for proliferation [39,47]. Fifth, this increased glycolytic character may provide tumor protection and enhance invasion; the low pH resulting from lactic acid accumulation may sensitize normal cells to invasion while protecting the tumor from the immune system [39,47]. Synergistically, these biological motivations for increasing glycolysis provide a survival advantage for a cancer cell.

As noted above, many glycolytic proteins are HIF targets, HIF is activated by many factors independent of oxygen levels, and HIF has been established to contribute to both the Pasteur and Warburg effects. Due to the fluctuating oxygenation states within a tumor it is possible that hypoxia induced glycolysis (the Pasteur effect) may pre-condition cancer cells for aerobic glycolysis (the Warburg effect).

**Figure 1:**
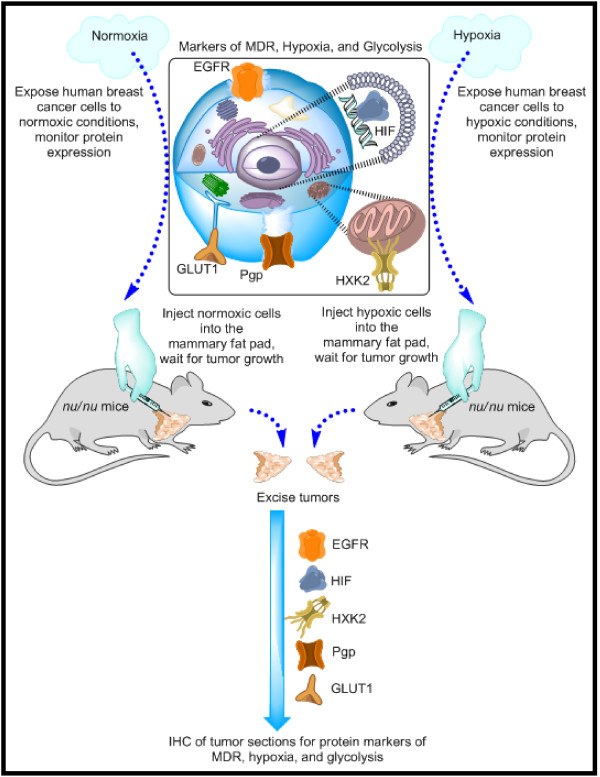
**Experimental Schema**. The primary aim of the current study was the development of an orthotopic model of multidrug resistant (MDR) breast cancer. To achieve that aim we conducted a study with two phases, *in vitro *and *in vivo*, as portrayed by the top and bottom portions of the figure. The first phase of the study consisted of exposing a panel of human cancer cells to either normoxic or hypoxic conditions and measuring the expression of protein markers for MDR, hypoxia, and glycolysis. These markers are portrayed by the cell diagram in the top, middle segment of the figure. The second phase of the study entailed selecting one cell line, exposing the human breast cancer cells to either normoxic or hypoxic conditions for five days, and then xenografting these cells into the mammary fat pad of nude mice. After tumors grew to 100 mm^3^, 250 mm^3^, and 500 mm^3^, they were excised and immunohistochemistry (IHC) was used to assess the expression of MDR, hypoxic, and glycolytic markers. Hypoxic pre-conditioning of xenografted cells did result in tumors with more MDR character than tumors established from normoxic cells. EGFR, epidermal growth factor receptor; HIF, hypoxia inducible factor; HXK2, hexokinase 2; Pgp, P-glycoprotein; GLUT1, glucose transporter 1.

As demonstrated by the schema in Figure 1, the first aim of the current study was to explore the relationship between hypoxia, MDR, and glycolysis in a panel of breast and ovarian cancer cell lines (top half of Figure 1). This was done by exposing the panel of cells to normoxic and hypoxic conditions, extracting the nucleic and basal protein, and examining protein expression through western blotting. Proteins included markers of MDR (Pgp and EGFR), markers of hypoxia (HIF-1α), and glycolytic markers (GLUT1 and HXK2); additional protein markers were also examined. Also demonstrated by the schema in Figure 1, the second aim of this study was to compare the protein expression of MDR, hypoxic, and glycolytic markers in tumor xenografts established from cells pre-exposed to normoxic conditions and from xenografts established from cells pre-exposed to hypoxic conditions (bottom portion of Figure 1). This was done through immunohistochemistry of the excised tumors. Within the two xenograft groups (normoxic pre-exposure and hypoxic pre-exposure), tumors of three different sizes were examined (100 mm^3^, 250 mm^3^, and 500 mm^3^). This study resulted in the establishment of an orthotopic model for MDR breast cancer.

## 2. Materials and methods

### 2.1. Cell Culture and Hypoxia

For this study, a panel of ovarian and breast cancer cell lines were selected. An established MDR ovarian cancer cell line, SKOV3_TR _cells, were used as a positive control for MDR protein character. Corresponding wild-type and hypoxic SKOV3 cells were also used. MDA-MB-231 breast cancer cells, were selected for their aggressive character. These cells are triple negative (negative for estrogen receptors, progesterone receptors, and HER2; human epidermal growth factor receptor 2). Triple negative breast cancer is known to be one of the most aggressive, recurrent multiforms of breast cancer. Another ovarian cancer cell line, OVCAR5 cells, were selected as a less aggressive comparison to the other cell types. MDA-MB-435 cells were selected as a negative control for EGFR expression.

SKOV3 cells, MDA-MB-231 cells, and OVCAR5 cells were obtained from ATCC (Manassas, VA). The SKOV3_TR _cells and the MDA-MB-435 cells were a kind gift from Dr. Duan (Massachusetts General Hospital, Sarcoma Molecular Biology Laboratory). Cells were plated at very low density and incubated at 37°C and maintained in RPMI-1640 media (Mediatech, Inc; Manassas, VA) supplemented with 10% fetal bovine serum (Gemini Bio-products; West Sacramento, CA) and 1% penicillin/streptomycin/amphotericin B mixture (Lonza; Walkersville, MD). To create hypoxic conditions using low-oxygen gas; cell culture flasks were placed in a modular incubation chamber (Billups-Rothenberg, Inc.; Del Mar, CA), flushed with a 0.5% O_2_, 5% CO_2_, nitrogen balanced gas for five minutes, and incubated at 37°C for various time points. As per manufacturer recommendations, the chamber was filled at a rate of 20 liters/minute for five minutes for complete saturation.

### 2.2. Protein Extraction and Western Blot Analysis

Basal and nucleic protein fractions were extracted from cells grown to 90% confluency in 75 cm^2 ^tissue culture flasks under normoxic and hypoxic conditions. Before extraction, cells were microscopically examined to confirm cell viability and the absence of excessive cell death. Basal protein was extracted using a high salt lysis buffer at 4°C. Nucleic protein was obtained using the NE-PER Nuclear and Cytoplasmic Extraction kit (Pierce Biotechnology; Rockford, IL). Protein concentrations were quantified using the BCA Protein Assay (Pierce Biotechnology). Protein was separated on 4-20% gradient SDS-PAGE gels (PAGEgel, Inc.; San Diego, CA) and transferred onto PVDF membranes (0.45 μm pore; Millipore, Billerica, MA). Membranes were blocked for 30 minutes with StartingBlock™ buffer (Pierce Biotechnology) before an overnight incubation with the primary antibody at 4°C. Membranes were then washed with TBST for 10 minutes (three times) and subsequently incubated with a horseradish peroxidase conjugated secondary antibody for 1 hour. Membranes were again washed with TBST, flash rinsed with deionized distilled water, incubated for 2-10 minutes in an enhanced chemiluminescence substrate (Pierce Biotechnology), and imaged using a Kodak FX Imaging Station (Rochester, NY). All blocking and washing steps were conducted at room temperature. P-glycoprotein antibody was purchased from Calbiochem while the EGFR antibody was purchased from Cell Signaling Technology (Danvers, MA). The remaining primary and secondary antibodies were purchased from Abcam (Cambridge, MA).

### 2.3. Protein Quantification

**Figure 2:**
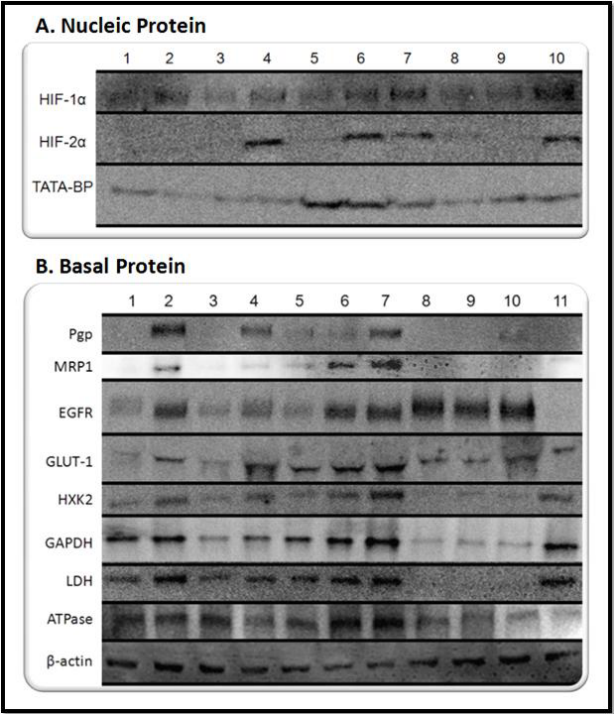
**Protein Expression Analysis**. Nucleic protein (A) and basal protein (B) were extracted from the panel of cell lines grown under normoxic (wild-type; WT) and hypoxic conditions (three and five days of hypoxia; 3-day Hyp and 5-day Hyp). The nuclear protein was probed for expression of the hypoxia inducible transcription factors HIF-1α and HIF-2α (TATA-binding protein was used as a nuclear loading control). Basal protein was probed for expression of MDR markers (P-glycoprotein, Pgp; multidrug resistance protein 1, MRP1), EGFR, glycolytic proteins (GLUT-1 glucose transporter; Hexokinase 2, HXK2; glyceraldehyde-3-phosphate dehydrogenase, GAPDH; lactate dehydrogenase, LDH), and mitochondrial ATP synthase. β-actin was used as a loading control for basal protein.

Semi-quantitative analysis was performed on the western blot data presented in Figure 2. To this end, Image J software was used; the integrated density of each band was measured, this value was divided by the integrated density of the control band to determine the relative intensity. The control band for nucleic protein expression was TATA-binding protein while the control band for basal protein expression was β-actin.

### 2.4. Animals and Orthotopic Model Development

Female *nu/nu *mice were procured from Charles River Laboratories (Wilmington, MA) and were housed in sterile cages on a 12:12 light/dark cycle with ad libitum acess to food and water. All procedures were approved by the Northeastern University Animal Care and Use Committee.

A total of 36 mice were used for this study; 18 received tumor cells pre-exposed to normoxic conditions for five days and 18 received tumor cells pre-exposed to hypoxic conditions for five days. Each group (normoxic and hypoxic) was further dived into three subgroups of 6 mice based on tumor size (100 mm^3^, 250 mm^3^, and 500 mm^3^).

To establish the xenografts, approximately 2 million human breast cancer cells (normal MDA-MB-231 or hypoxic MDA-MB-231 cells) suspended in a 100 μl of a 50:50 mix of matrigel and serum free medium was injected into the mammary fat pad of the mice while they were under light isoflurane anesthesia. Pre-chilled, sterile syringes with 27 gauge, ½'' needles were used to inject the tumor cells. Syringes were pre-chilled at 4°C to prevent coagulation and immediate gelling of the matrigel.

The tumor size was measured every other day using Vernier calipers in two dimensions. Individual tumor volumes were calculated using the formula volume = [length × (width)^2^]/2 where length is the longest diameter and width is the shortest diameter perpendicular to length. Tumors were allowed to grow to the allocated volumes of 100 mm^3^, 250 mm^3^, and 500 mm^3^. During this time, animals were monitored every alternate day for body weight, eating/drinking behavior, and general health. Once tumors reached the desired size, the mice were euthanized via carbon dioxide inhalation. Tumors were then excised.

### 2.5. Immunohistochemistry of Tumors

Excised tumors were embedded in section medium (Richard-Allan Neg 50*, Thermo Scientific, Waltham, MA), flash frozen in liquid nitrogen, and stored at -80°C until use. Embedded tumors were thawed to -20°C, cryo-sectioned into 7 μm thick sections, and mounted onto glass slides (SuperFrost Plus^®^, Thermo Scientific, Waltham, MA). Sections were outlined with an Aqua Hold Pap Pen (Scientific Device Laboratory, Des Plaines, IL), air dried at room temperature, then stored at -20°C. Sections were then thawed to room temperature and fixed in ice-cold acetone for 10 minutes. Sections were air dried at room temperature for 1 hour, and rinsed in two changes of cold PBS (5 minutes each). Then sections were incubated with 100 μl of IHC Select^® ^Blocking Reagent (Chemicon, Billerica, MA) in a humidified chamber at 37°C for 30 minutes. The blocking buffer was then drained off, the slides rinsed in PBS, and then each section was incubated with 100 μl of primary antibody diluted in IHC Select^® ^Antibody Diluent Solution (Chemicon, Billerica, MA), overnight at 4°C. Slides were rinsed in two changes of PBST and each section was incubated with 100 μl of secondary antibody diluted in IHC Select^® ^Antibody Diluent Solution (Chemicon, Billerica, MA), at room temperature for 30 minutes. Slides were washed in two changes of PBS and incubated with a solution of Alexa Fluor^® ^568 phalloidin (to stain F-actin) and Hoechst 33342 (to stain nuclei) (Invitrogen; Carlsbad, CA) for 20 minutes. Slides were rinsed in PBST and dehydrated in 95% ethanol for 2 minutes, and 100% ethanol for two exchanges (3 minutes each).

Tissue sections were then immersed with Prolong Gold^® ^Antifade reagent (Invitrogen; Carlsbad, CA), covered with glass cover slips, and allowed to cure overnight at room temperature. All primary antibodies were from Cell Signaling Technology (Danvers, MA), except for the GLUT-1 antibody which was from Abcam (Cambridge, MA). The secondary antibodies were Alexa Fluor^® ^488 goat anti-mouse IgG (H + L) and Alexa Fluor^® ^488 goat anti-rabbit IgG (H + L) (Invitrogen; Carlsbad, CA). Slides were imaged using an Olympus IX51 Microscope.

## 3. Results

### 3.1. Protein Analysis of Hypoxic, MDR, and Glycolytic Markers

The panel of cell lines (detailed in the materials and methods) were exposed to hypoxic conditions (0.5% oxygen) for three and five days. The basal protein and nucleic protein from cells exposed to hypoxic and normoxic conditions was extracted and western blotting was used to analyze the expression of hypoxic factors, MDR markers, and downstream proteins induced under hypoxic regulation.

Nuclear protein analysis (Figure 2A) revealed that HIF-1α expression was apparent in all cells but was elevated in the MDR cells (lane 2), in cells exposed to hypoxic conditions for 3 days (lanes 3, 6, and 9), and in cells exposed to hypoxic conditions for 5 days (lanes 4, 7 and 10). Interestingly, in the SKOV3 cell line (lane 3) and in the OVCAR5 cell line (lane 9), 3-days of hypoxia was not substantial in inducing HIF-2α nuclear translocation (Figure 2A). Yet, in the MDA-MB-231 breast cancer cells (lane 6), 3 days of hypoxia did induce nuclear translocation of HIF-2α. Five days of hypoxic exposure resulted in nuclear accumulation of HIF-2α in all three cell lines (lane 4, 7, and 10). Also of significance, HIF-2α nuclear translocation was not evident in the MDR cells.

Markers of MDR were also examined in the three cell lines under normoxic and hypoxic conditions (Figure 2B). The high expression of Pgp in the MDR cells is evident (lane 2). Three days of hypoxic exposure was not sufficient to induce Pgp expression in the SKOV3 cells (lane 3) or to increase Pgp expression in the MDA-MB-231 cells (lane 6, which appears the same as the basal level in lane 5). Five days of hypoxia, however, was sufficient in inducing Pgp expression in both the SKOV3 ovarian cancer cells (lane 4) and in the MDA-MB-231 breast cancer cells (lane 7). The OVCAR5 ovarian cancer cells appear more resistant to Pgp induction as there is only a faint band of protein after five days of hypoxic exposure (lane 10). The only wild type cancer cell line that expresses a basal level of Pgp under normoxic conditions is the MDA-MB-231 breast cancer cell line (lane 5). MRP-1 (multi-drug resistance protein 1) is also an ABC transporter involved in drug efflux. The MRP-1 expression profile was similar to the expression of Pgp in the cell lines examined. The only significant difference in MRP-1 expression is that 3 days of hypoxic exposure was sufficient in increasing MRP-1 expression in the MDA-MB-231 cells (lane 6) while five days of hypoxia did not even induce faint expression in the OVCAR5 cells (lane 10).

Although over-expression of growth factors is characteristic of cancer cells in general, this over-expression is a particularly vital survival mechanism for hypoxic MDR tumor cells as these cells are often distal from a constant supply of nutrients and growth factors; hypersensitivity to these factors increases the propensity for growth and maintenance. As expected, EGFR expression was elevated in MDR cells (lane 2) relative to wild type SKOV3 cells maintained under normoxic conditions (lane 1) (Figure 2B). Three days of hypoxic exposure did not substantially increase EGFR expression in SKOV3 cells (lane 3), yet five days of hypoxic exposure resulted in a more pronounced expression level (lane 4). For the MDA-MB-231 breast cancer cells, there was a marked increase in EGFR expression after both 3 and 5 days of hypoxic exposure. The high basal level of EGFR expression in OVCAR5 cells grown under normoxic conditions (lane 8) was maintained after 3 days of hypoxia (lane 9) and elevated after 5 days of hypoxia (lane 10). Wild type MDA-MB-435 cells grown under normoxic conditions do not express notable levels of EGFR (lane 11). As with other HIF-1α targets, the level of EGFR induction seems to be cell type dependent and related to a threshold of hypoxic exposure. TATA-binding protein was used as a loading control for nucleic protein (Figure 2A) and β-actin was used as a loading control for basal protein (Figure 2B).

To examine the relationship between hypoxia, MDR, and glycolysis the protein expression of four glycolytic proteins that are transcriptionally activated by HIF-1α were analyzed in the panel of cells; GLUT-1 glucose transporter, Hexokinase 2 (HXK2), glyceraldehyde-3-phosphate dehydrogenase (GAPDH), and lactate dehydrogenase (LDH) (Figure 2B).

GLUT-1 glucose transporter is significant to MDR hypoxic cell survival in much the same way as EGFR. These cells that are located in poorly or non-vascularized regions are starved for nutrients and increase their prospects of survival by increasing the expression of nutrient importers such as GLUT-1. As demonstrated in Figure 2B, MDR cells (lane 2) have an elevated level of GLUT-1 expression relative to wild type SKOV3 cells grown under normoxic conditions (lane 1). Three days of hypoxia was not sufficient in elevating this expression in SKOV3 cells (lane 3) and OVCAR5 cells (lane 9), but elevation was evident after five days of hypoxia (lane 4 and lane 10). Conversely, three days of hypoxia appeared to slightly increase GLUT-1 in MDA-MB-231 cells (lane 6) while five days of hypoxia further increased this expression (lane 7).

The expression of hexokinase 2 (the enzyme that catalyzes the first step of glycolysis) was similar to the expression of GAPDH (an intermediate glycolytic enzyme) and LDH (the terminal glycolytic enzyme) (Figure 2B). For these three proteins, there was only a slightly higher expression level in the MDR cells (lane 2) relative to normoxic SKOV3 cells (lane 1) while increased hypoxic exposure lead to an increase in protein expression in the SKOV3 cell line and the MDA-MB-231 cell line. The OVCAR5 cells appeared to have a low basal level of HXK2, GAPDH, and LDH that did not appear to be induced by hypoxia (lanes 8, 9, and 10). Conversely, the MDA-MB-435 wild type cells grown under normoxic conditions express high basal levels of HXK2, GAPDH, and LDH (lane 11).

Interestingly, expression of mitochondrial ATP synthase, the ATP producing component of OXPHOS coupled to the electron transport chain, did not correlate with hypoxic exposure across the cell lines. There was no difference in expression between the MDR cells and the normoxic SKOV3 cells while five days of hypoxia decreased expression in this cell line. Five days of hypoxia also decreased expression in the OVCAR5 cell line. On the other hand, hypoxia (three and five days) increased the expression of ATP synthase in the MDA-MB-231 cell line.

**Figure 3:**
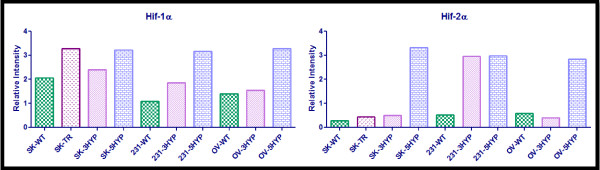
**Semi-quantitative Analysis of HIF-1α and HIF-2α**. Image J software was used to determine the relative intensity of protein expression. The integrated density of each band was measured; this value was divided by the integrated density of the TATA-binding protein band to determine the relative intensity. All wild-type, normoxic (WT) cell lines are indicated by checkered green bars, all cell lines exposed to 3-days hypoxia (3HYP) are indicated by diagonal-lined pink bars, all cell lines exposed to 5-days of hypoxia (5HYP) are displayed by blue brick bars, and the established MDR cell line (TR) is displayed as the speckled purple bar (the second bar on each graph). The cell lines are SKOV3 (SK), MDA-MB-231 (231), and OVCAR5 (OV).

**Figure 4:**
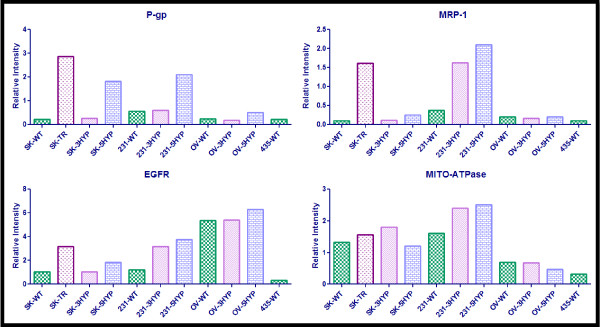
**Semi-quantitative Analysis of P-gp, MRP-1, EGFR, and MITO-ATPase**. Image J software was used to determine the relative intensity of protein expression. The integrated density of each band was measured; this value was divided by the integrated density of the β-actin protein band to determine the relative intensity. All wild-type, normoxic (WT) cell lines are indicated by checkered green bars, all cell lines exposed to 3-days hypoxia (3HYP) are indicated by diagonal-lined pink bars, all cell lines exposed to 5-days of hypoxia (5HYP) are displayed by blue brick bars, and the established MDR cell line (TR) is displayed as the speckled purple bar (the second bar on each graph). The cell lines are SKOV3 (SK), MDA-MB-231 (231), OVCAR5 (OV), and MDA-MB-435 (435).

**Figure 5:**
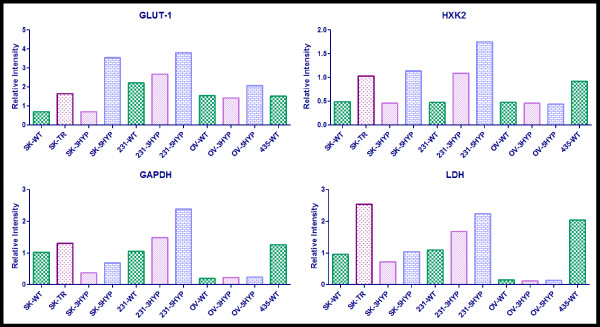
**Semi-quantitative Analysis of GLUT-1, HXK2, GAPDH, and LDH**. Image J software was used to determine the relative intensity of protein expression. The integrated density of each band was measured; this value was divided by the integrated density of the β-actin protein band to determine the relative intensity. All wild-type, normoxic (WT) cell lines are indicated by checkered green bars, all cell lines exposed to 3-days hypoxia (3HYP) are indicated by diagonal-lined pink bars, all cell lines exposed to 5-days of hypoxia (5HYP) are displayed by blue brick bars, and the established MDR cell line (TR) is displayed as the speckled purple bar (the second bar on each graph). The cell lines are SKOV3 (SK), MDA-MB-231 (231), OVCAR5 (OV), and MDA-MB-435 (435).

Semi-quantitative analysis was performed on the western blot data presented in Figure 2. The results of this analysis are presented in Figure 3, Figure 4, and Figure 5. The semi-quantitative analysis is consistent with the interpretations of the western blot data; hypoxic exposure appears to increase the expression of HIF-1α, HIF-2α, Pgp, MRP-1, EGFR, and the glycolytic proteins in the SKOV3 and MDA-MB-231 cells yet the OVCAR5 cells appear to be resistant to hypoxia induced MDR and hypoxia induced glycolysis.

### 3.2. Development and Characterization of MDR Tumor Xenografts

The MDA-MB-231 cells were selected for hypoxic pre-conditioning and tumor xenografting as these cells demonstrated the most notable response to hypoxia in the *in vitro *studies; hypoxia substantially increased the expression of MDR markers and glycolytic proteins relative to the expression in normoxic cells. A total of 36 mice were used for this study; 18 mice were injected with MDA-MB-231 cells grown under normoxic conditions while 18 were injected with MDA-MB-231 cells pre-exposed to hypoxic conditions. The cells were injected in the mammary fat pad of the mice for the development of orthotopic tumors. Tumors were grown to three sizes; 100 mm^3^, 250 mm^3^, and 500 mm^3^. The objectives of this study was to determine which group resulted in tumors with the most MDR character by assessing (1) if there was a difference between tumors developed from cells grown under normoxic conditions and tumors developed from cells pre-exposed to hypoxic conditions, (2) if there was a difference between tumors of different sizes. To determine such differences between the groups, 6 proteins were selected for tissue immunohistochemistry; Pgp, CD-31, HIF-1α, EGFR, HXK2, and GLUT-1. Tumors were sectioned into 7 uM slices, F-actin was labeled with Alexa Fluor^® ^568 phalloidin (red), nuclei were stained with Hoechst 33342 (blue), and the sections were probed for the protein of interest using primary antibodies against the protein and Alexa Fluor^® ^488 conjugated secondary antibodies (green).

**Figure 6:**
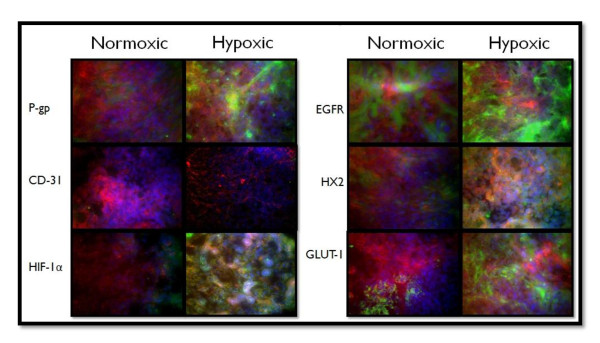
**Immunohistochemistry of 100 mm^3 ^Normoxic and Hypoxic Tumor Xenografts**. Tissue sections were probed with primary antibodies against the protein of interest, then labeled with Alexa Fluor^® ^488 conjugated secondary antibodies (green). F-actin was stained with Alexa Fluor^® ^568 phalloidin (red) and nuclei were stained with Hoechst 33342 (blue). These images represent protein expression in the tumor core.

**Figure 7:**
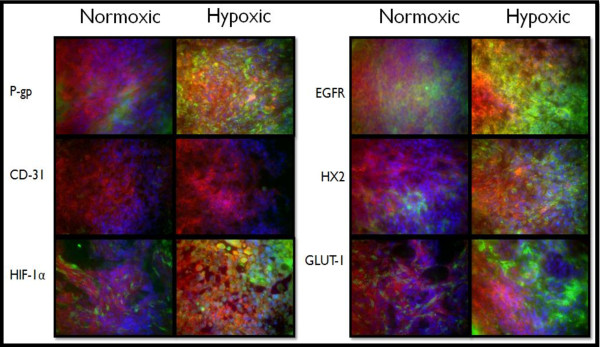
**Immunohistochemistry of 250 mm^3 ^Normoxic and Hypoxic Tumor Xenografts**. Tissue sections were probed with primary antibodies against the protein of interest, then labeled with Alexa Fluor^® ^488 conjugated secondary antibodies (green). F-actin was stained with Alexa Fluor^® ^568 phalloidin (red) and nuclei were stained with Hoechst 33342 (blue). These images represent protein expression in the tumor core.

**Figure 8:**
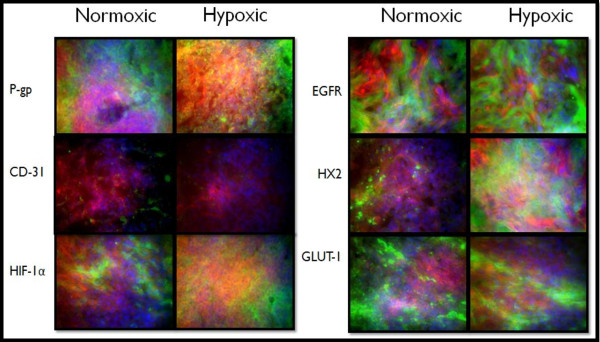
**Immunohistochemistry of 500 mm^3 ^Normoxic and Hypoxic Tumor Xenografts**. Tissue sections were probed with primary antibodies against the protein of interest, then labeled with Alexa Fluor^® ^488 conjugated secondary antibodies (green). F-actin was stained with Alexa Fluor^® ^568 phalloidin (red) and nuclei were stained with Hoechst 33342 (blue). These images represent protein expression in the tumor core.

The 100 mm^3 ^tumors are illustrated in Figure 6, the 250 mm^3 ^tumors are illustrated in Figure 7, and the 500 mm^3 ^tumors are illustrated in Figure 8. Figures 6, 7, &8 depict IHC of tumor cores representative of *n *= 6 for each group. There were demonstrated differences in the protein expression profiles of 100 mm^3 ^tumors developed from cells grown under normoxic conditions and from tumors developed from cells pre-exposed to hypoxic conditions. There was minimal and localized expression of Pgp in the tumors developed from normoxic cells whereas there was profuse expression of Pgp in the tumors developed from hypoxic cells. There was no apparent difference in CD-31 expression; this marker was used to assess angiogenesis. Again, there was localized and minimal expression of HIF-1α in the tumors developed from cells grown under normoxic conditions whereas there was profuse expression in the tumors derived from hypoxic cells; this expression appeared to be localized with both the cytoplasmic and nucleic fractions of the cells. Both groups of tumors had high levels of EGFR. Expression of hexokinase 2 and GLUT-1 was minimal and localized in the tumors derived from normoxic cells and profuse in the tumors derived from hypoxic cells.

These expression trends continued in the 250 mm^3 ^tumors (Figure 7), although there was less of a difference as the tumors developed from normoxic cells seem to express higher levels of these marker proteins in tumors of this size. Excessive expression of all marker proteins (except CD-31) was apparent in both groups of tumors grown to 500 mm^3 ^(Figure 8). The only substantial CD-31 expression was in the tumors derived from normoxic cells grown to 500 mm^3 ^(Figure 8).

**Figure 9:**
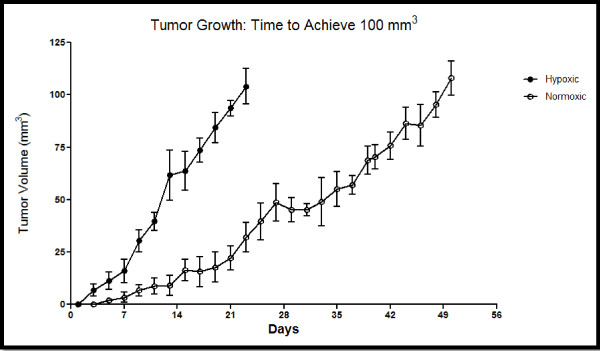
**Normoxic and Hypoxic tumor Growth; Time to Achieve 100 mm^3^**. Normoxic and Hypoxic tumor xenografts were established in the mammary fat pad of female nude mice and tumor growth was monitored until 100 mm^3 ^tumors were achieved. The tumor size was measured every other day using Vernier calipers in two dimensions. Individual tumor volumes were calculated using the formula volume = [length × (width)^2^]/2 where the length was the longest diameter and the width was the shortest diameter perpendicular to length. For each group, *n *= 6. Each data point represents the mean ± SD.

Based on these distinctions, the 100 mm^3 ^tumors developed from hypoxic cells appear to have the most MDR character that is clearly distinct from the tumors developed from normoxic cells. As the tumor size increased, the differences between the tumors derived from normoxic and hypoxic cells decreased. There was also a remarkable difference in the growth rate of tumors developed from normoxic cells and those developed from hypoxic cells (Figure 9). As demonstrated in Figure 9, the tumors developed from hypoxic cells reached a 100 mm^3 ^size within 3 weeks whereas it took between 7-8 weeks for the tumors developed from normoxic cells to reach this size. After reaching 100 mm^3^, the differences in the rates of tumor growth decreased as it took 2.5-3 months for both groups of tumors to reach 500 mm^3 ^(not shown).

**Figure 10:**
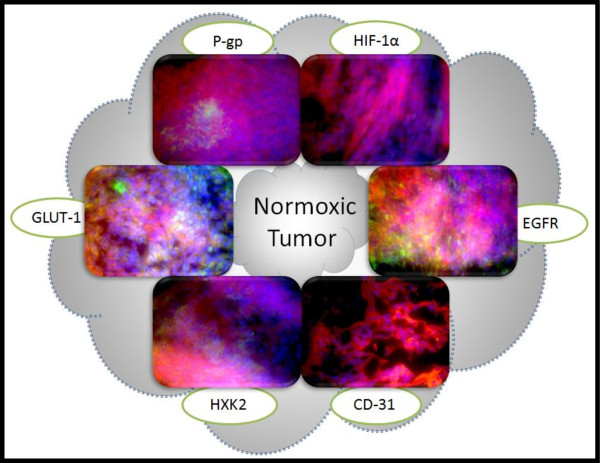
**Immunohistochemistry of 100 mm^3 ^Normoxic Tumor Perimeter**. Tissue sections were probed with primary antibodies against the protein of interest, then labeled with Alexa Fluor^® ^488 conjugated secondary antibodies (green). F-actin was stained with Alexa Fluor^® ^568 phalloidin (red) and nuclei were stained with Hoechst 33342 (blue). These images represent protein expression in the tumor perimeter.

**Figure 11:**
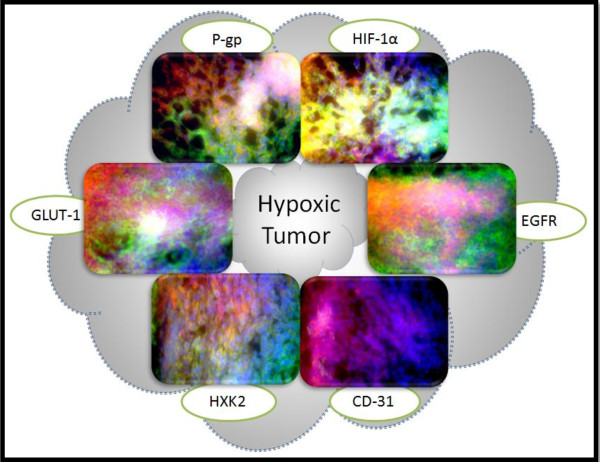
**Immunohistochemistry of 100 mm^3 ^Hypoxic Tumor Perimeter**. Tissue sections were probed with primary antibodies against the protein of interest, then labeled with Alexa Fluor^® ^488 conjugated secondary antibodies (green). F-actin was stained with Alexa Fluor^® ^568 phalloidin (red) and nuclei were stained with Hoechst 33342 (blue). These images represent protein expression in the tumor perimeter.

To further examine the distinctions between the 100 mm^3 ^tumors developed from normoxic cells and those developed from hypoxic cells, IHC of the tumor perimeter was examined (Figure 10 and Figure 11). (Figures 6, 7, and 8 represent the tumor cores). There is a consistent difference between the expression of these proteins in the core (Figure 6) and perimeter (Figure 10 and 11) of tumors developed from normoxic cells and in those developed from hypoxic cells.

As with the tumor core, there is a low level of Pgp expression in the perimeter of tumors developed from normoxic cells. On the other hand, Pgp expression seemed higher in the perimeter of tumors developed from hypoxic cells than in the core. Hif-1α expression was also higher in the perimeter of tumors derived from hypoxic cells than in the core. Hif-1α expression was low in the core of tumors derived from normoxic cells and non-detectable in the perimeter. EGFR expression appeared to be decreased in the perimeter of tumors grown from normoxic cells relative to the core. Conversely, EGFR expression maintained high expression levels in both the perimeter and core of tumors developed from hypoxic cells. There was no apparent difference in CD-31 expression between the perimeter and core of both groups of tumors as all sections had either very low or non-detectable levels of this protein. The expression of hexokinase 2 was consistent between the tumor perimeter and tumor core for both groups of tumors; with low and localized levels in the tumor sections developed from normoxic cells and highly profuse expression in the tumor sections developed from hypoxic cells. GLUT-1 expression was high in both the tumor perimeter and core of tumor sections derived from hypoxic cells whereas the perimeter of tumors developed from normoxic cells had higher and more profuse expression than the core where expression was very localized.

Collectively, the expression of Pgp and Hif-1α was elevated in the perimeter of tumors established from hypoxic cells relative to the core while the perimeter of tumors established from normoxic cells had lower expression of EGFR and higher expression of GLUT-1 relative to the tumor core. Based on the differences in protein expression between the groups of tumors in both the perimeter and the core, as well as the differences in the early growth rates of the tumors, it was inferred that 100 mm^3 ^tumors developed from hypoxic cells have more MDR character than 100 mm^3 ^tumors developed from normoxic cells. These tumors established from hypoxic pre-conditioned cells may be a useful model for the study of MDR breast cancer.

## 4. Discussion

### 4.1. Inclusion of MDA-MB-435 Cells

Although the lack of EGFR expression is well characterized in the MDA-MB-435 cell line, the actual origin of this cell line is controversial. Recent studies suggest that this cell line has been incorrectly characterized as a breast carcinoma cell line and is more likely derived from melanocytes [48]. Regardless of the controversy, this cell line was included in these experiments as a negative control for EGFR-expression.

### 4.2. Protein Changes in Response to Hypoxia

Expression of active HIF-1α in the nucleus of cells grown under normoxic conditions is most likely a cellular response to previously mentioned oxygen-independent factors (such as EGFR or phosphatidylinositol 3-kinase), or a response to stress factors and homeostatic dis-regulation that is characteristic of cancer. For example, von Hippel-Lindau is a tumor suppressor protein that contributes to the transformation of certain types of cancer, such as renal cell carcinoma, via negative regulation of HIF-1α [22,49,50].

An interesting distinction between HIF-1α and HIF-2α expression was that HIF-1α expression was apparent in all cell lines whereas HIF-2α appeared only to be induced by hypoxia. This suggests that hypoxia induces the nuclear translocation of HIF-2α, yet oxygen-independent factors and conditions of cell stress (such as those in the MDR cells) are not sufficient to induce this translocation. Based on these results, it is likely that both HIF-1α and HIF-2α isoforms are central to the development of MDR but induction of each isoform occurs in response to different cellular and environmental factors. This could result in different MDR phenotypes as the targets of each isoform vary.

Increased glycolysis in the absence and presence of oxygen (the Pasteur and Warburg effects) are important hallmarks of cancer. The Warburg Effect is associated with aggressive, MDR cancer [4,21,29,51,52]. Glycolysis is another thread that connects MDR and hypoxia; increased glycolysis is characteristic of MDR cancer and most glycolytic enzymes are HIF-1α targets that are transcriptionally activated in response to hypoxia [21,22,24,26,28,29,32-36]. Activation of HIF-1α by glycolytic proteins has also been demonstrated, suggesting a feedback loop between the glycolytic and HIF-1α pathways [34]. However, as these results demonstrate, cancer cells do not have a universal response to hypoxia. The Pasteur effect appears to be duration dependent in the SKOV3 and MDA-MB-231 cells yet the OVCAR5 cells seem resistant to hypoxia induced glycolysis. The feedback loop between hypoxia and glycolysis may be more relevant in certain subsets of cancer and non-existent in other types of cancer.

The hypoxic transformation, phenotype changes in response to hypoxia, do not appear to be consistent among the cell lines in regard to mitochondrial ATP synthase expression. The hypoxic transformation of MDA-MB-231 cells may lead to such an excessive demand for energy that the expression of both glycolytic and OXPHOS enzymes is increased as a survival response. Hypoxic transformation of SKOV3 cells may depress the cell cycle, decreasing the energetic demands of cell, leading to decreased OXPHOS. It is possible also that these differences in ATP synthase expression correlate more to mitochondrial differences between the cell lines than to a hypoxic response; a more complete profiling of mitochondrial enzymes would be necessary to determine this.

Collectively, the protein analysis results indicate that each cell line has a cell-specific threshold for hypoxic transformation. The MDA-MB-231 breast cancer cells underwent the most significant hypoxic transformation with an increase in all proteins examined (HIF transcription factors, HIF targets, MDR proteins, and glycolytic proteins). Although hypoxia induced MDR character and glycolysis in the SKOV3 ovarian cancer cells, the transformation required more chronic hypoxic exposure (five days). Five days of hypoxia was not sufficient in transforming the OVCAR5 cells. These results demonstrate the diverse proteomic responses of the different cell lines. Although the OVCAR5 cells express GLUT-1, these cells appear to have less overall glycolytic character than the other cells examined (lower HXK2, GAPDH, and LDH) and appear to be resistant to hypoxia induced MDR and hypoxia induced glycolysis. These cells may require additional stimuli for hypoxic transformation or may have a genetic resistance to the hypoxia induced HIF cascade. These results also suggest that the distinction between the Pasteur and Warburg effects may not be universal as the degree of hypoxia (percent oxygen depletion) is critical in determining the cell specific response. The Pasteur effect is a suppression of glycolysis in the presence of oxygen whereas the Warburg effect is glycolysis in the presence of oxygen. The current results reveal that there are cell-specific thresholds for the Pasteur effect; additional and more detailed studies would likely reveal a critical oxygen concentration for each cell type where the Pasteur and Warburg effects are difficult to distinguish. Perhaps more significant than the isolated study of the Pasteur and Warburg effects is the unfolding relationship between HIF and glycolysis as this relationship is present in cancer cells under both normoxic and hypoxic conditions [34,35,39-41].

Although unlikely, as cells were plated at a low density, it is important to note that as the oxygen consumption rate was not measured in these studies, it is possible that peri-cellular oxygen depletion in the normoxic cell population could create hypoxic conditions, which would make the expression profile of normoxic cells more similar to hypoxic cells.

### 4.3. Orthotopic Model Development

An important finding of this study was that the protein expression profile of both tumor xenografts developed from normoxic cells and xenografts developed from hypoxic cells changed as the tumor size increased. The 100 mm^3 ^xenografts established from hypoxic cells were clearly distinguishable from the 100 mm^3 ^xenografts established from normoxic cells, but these differences decreased as the tumor size increased.

The elevated level of HIF-1α in the perimeter of 100 mm^3 ^tumors developed from hypoxic cells is less likely due to lower oxygen relative to the core (as it is probable that the perimeter is more vascularized) and more likely due to oxygen-independent factors, cell stressors, oncogenes, and tumor suppressors that characterize cancer and up-regulate HIF. The elevated level of Pgp in the perimeter of tumors (100 mm^3^) developed from hypoxic cells relative to the core may be due to the increased expression of HIF, as Pgp is transcriptionally activated by HIF, or may be a spatial response to microenvironmental factors as it is these cells in the perimeter of a tumor that are most likely to have the first contact with therapeutic agents. The decreased EGFR expression in the perimeter of tumors established from normoxic cells relative to the core may be because the core of the tumor is more starved for growth factor activation, and hence increases EGFR in an attempt to be more susceptible to growth factors. Alternatively, the higher level of GLUT-1 in the perimeter of tumors derived from normoxic cells relative to the core may be due to a positive feedback loop with available glucose. Collectively, the IHC analysis of the tumor cores and perimeters (as well as the growth curves) illustrate the more pronounced MDR character of the 100 mm^3 ^tumors established from hypoxic cells relative to 100 mm^3 ^tumors developed from normoxic cells.

Hypoxic pre-conditioning of the tumor cells may result in more aggressive, MDR xenografts due to the upregulation of HIF and subsequent activation of HIF targets (such as growth factor receptors, MDR efflux pumps, and glycolytic proteins); this was demonstrated by the western blot data in this study. This activation may provide these cells with an initial growth advantage during xenografting establishment, as demonstrated by the tumor growth curves. The hypoxic pre-conditioning may activate the HIF cascade and MDR character in the cells, enabling initial, rapid growth of the xenografted cells before the establishment of profuse tumor vasculature, whereas the xenografts established from normoxic cells may not exhibit aggressive phenotypes until the angiogenic process is complete. This correlates with the expression of CD-31 (angiogenic marker), as the only substantial CD-31 expression was seen in the tumor sections of 500 mm^3 ^xenografts established from normoxic cells. Expression of CD-31 in the 500 mm^3 ^xenografts established from normoxic cells coincided with an increase in expression of HIF and all down-stream targets of HIF associated with MDR, similar to the expression of HIF, Pgp, EGFR, HXK2, and GLUT-1 in the the 500 mm^3 ^xenografts established from hypoxic cells. Angiogenesis has long been associated with primary tumor growth and metastasis, but recent evaluations of anti-angiogenic therapy reveal that anti-angiogenic therapies can actually increase metastasis and invasion [53-55]. These studies suggest hypoxic tolerance as a possible mechanism for this increased invasion and metastasis [54]. Hypoxic pre-conditioning may elicit a similar cellular response as is seen with anti-angiogenic therapy, resulting in aggressive disease.

Exploiting the Pasteur effect by using hypoxic pre-conditioning is a useful technique for creating MDR tumor xenografts. We have developed an animal model of MDR breast cancer using hypoxic MDA-MB-231 cells. Although this approach of using hypoxic cells for xenografts will not be effective for every type of cancer, as demonstrated with the OVCAR5 cells, it will undoubtedly be applicable to other cancer cell lines. This approach could dramatically expand the repertoire of MDR cancer models and could be used to study the Pasteur and Warburg effects in orthotopic models. Using this method of hypoxic pre-conditioning could enable the rapid *in vivo *study of many different types of MDR cancer, which would not otherwise be possible.

## 5. Conclusions

Hypoxia alone is sufficient in transforming normal cancer cells into MDR cancer. Hypoxia also induces glycolysis in some cancer cells (the Pasteur effect) further increasing their survival advantage. Increased expression of MDR and glycolytic proteins in response to hypoxia is cell-type and duration dependent.

An animal model was developed for orthoptopic MDR breast cancer using hypoxic MDA-MB-231 cells in female athymic mice. Immunohistochemistry of the tumor model demonstrated that the tumors derived from hypoxic cells had higher MDR character relative to tumors derived from normoxic cells. This character was maintained in the tumor perimeter and core. The tumors established from hypoxic cells also demonstrated more aggressive growth relative to tumors established from normoxic cells. This method of hypoxic pre-conditioning could be used to develop a multitude of orthotopic animal models of MDR cancer, further expanding the study of the disease.

## 6. Competing interests

The authors declare that they have no competing interests.

## 7. Authors' contributions

LM carried out the experiments and drafted the manuscript. LM, ZD and MA participated in the design of the study. All authors have read and approved the final manuscript.
